# Fortified interpenetrating polymers – bacteria resistant coatings for medical devices†
†Electronic supplementary information (ESI) available: Supporting figures and tables, and additional experimental procedures. See DOI: 10.1039/c6tb01110a



**DOI:** 10.1039/c6tb01110a

**Published:** 2016-07-18

**Authors:** Seshasailam Venkateswaran, Orlando David Henrique Dos Santos, Emma Scholefield, Annamaria Lilienkampf, Peter J. Gwynne, David G. Swann, Kevin Dhaliwal, Maurice P. Gallagher, Mark Bradley

**Affiliations:** a EaStCHEM School of Chemistry , University of Edinburgh , King's Buildings , West Mains Road , Edinburgh , EH9 3FJ , UK . Email: mark.bradley@ed.ac.uk; b Laboratório de Fitotecnologia , Departamento de Farmácia , Escola de Farmácia , Universidade Federal de Ouro Preto , Ouro Preto , Minas Gerais 35400-000 , Brazil; c MRC Centre for Inflammation Research , The Queens Medical Research Institute , University of Edinburgh , 47 Little France Crescent , Edinburgh EH16 4TJ , UK; d School of Biological Sciences , University of Edinburgh, King's Buildings , West Mains Road , Edinburgh , EH9 3JF , UK; e Critical Care , NHS Lothian , Royal Infirmary of Edinburgh , 51 Little France Crescent , Edinburgh , EH16 4SA , UK

## Abstract

Infections arising from contaminated medical devices are a serious global issue, contributing to antibiotic resistance and imposing significant strain on healthcare systems. Since the majority of medical device-associated infections are biofilm related, efforts are being made to generate either bacteria-repellent or antibacterial coatings aimed at preventing bacterial colonisation. Here, we utilise a nanocapsule mediated slow release of a natural antimicrobial to improve the performance of a bacteria repellent polymer coating. Poly(lauryl acrylate) nanocapsules containing eugenol (4-allyl-2-methoxyphenol) were prepared and entrapped within a interpenetrating network designed to repel bacteria. When coated on a catheter and an endotracheal tube, this hemocompatible system allowed slow-release of eugenol, resulting in notable reduction in surface-bound *Klebsiella pneumoniae* and methicillin resistant *Staphylococcus aureus.*

## Abstract 2

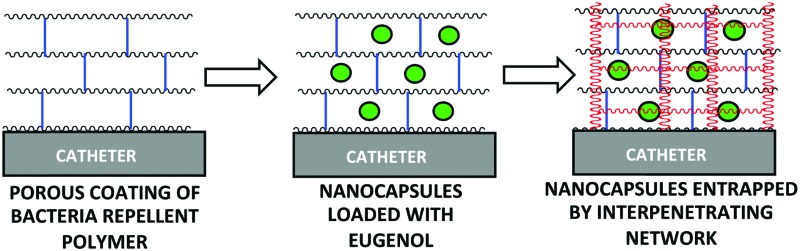
Nanocapsule-mediated eugenol release from an interpenetrating polymer network coating reduces bacterial binding on medical devices.

## Introduction

Polyurethanes, polysilicones and polyvinyl chloride are widely used in the manufacture of medical devices due to their biocompatibility and good mechanical properties.^1^ However, microorganisms can adhere to these polymeric surfaces, resulting in medical devices being one of the most common sources of nosocomial infections.^2^ Once bacteria attach to the surface, they multiply and subsequently form a biofilm (matrix composed of exopolysacharides, proteins, teichoic acids and extracellular DNA), which is a source of a chronic infection.^3–5^ Bacterial biofilms are inherently resistant to antimicrobials^5^ and, aided by the protection of biofilms and limited diffusion,^4^ bacteria may be exposed to sub-inhibitory concentrations of antibiotics during treatments, promoting the development of antibiotic resistance. Therefore in an effort to reduce biofilm formation, coatings have been developed for medical devices that will either inhibit attachment of bacteria or kill bacteria on contact.^1^ Coatings to reduce bacterial binding include hydrophilic polymers such as poly(ethylene glycol) (PEG),^6^ peptide–PEG amphiphilic macromolecules,^7^ pluronic surfactants,^8^ zwitterionic polyurethanes,^9^ and super hydrophobic coatings.^10^ Previously, we have reported the high-throughput identification of bacteria repelling polymers,^11,12^ with poly(methyl methacrylate-*co*-dimethylacrylamide) (PA13) in particular demonstrating reduced binding of several clinical isolates on coated medical devices.^12^ Coatings that inherently reduce bacterial attachment are an attractive strategy; however, *in situ* formation of layers of bacterial and host components on the device surface promotes bacterial attachment reducing their effectiveness.^1^ Hence, a bacteria repellent coating that also incorporates antibacterial components that inhibit biofilm formation offer the potential to be more effective.^13^

Clove oil and its major ingredient eugenol (4-allyl-2-methoxyphenol) have been studied as broad-spectrum antimicrobials with various mechanisms of action proposed.^14–16^ FDA approved eugenol (used in dentistry as an analgesic) has been reported to inhibit formation of biofilms of *Klebsiella pneumoniae* (*K. pneumoniae*),^17^ *Staphylococcus aureus* (*S. aureus*)^18^ and *Candida albicans* ^19^ at sub-inhibitory concentrations without promoting resistance due to its multi-target mode of action.^20^ Reduced expression of virulence-related exoproteins^21^ and down-regulation of biofilm-associated genes^22^ by eugenol have also been demonstrated. At sub-inhibitory concentrations, eugenol interacts synergistically with various antibiotics (*e.g.* penicillin, ampicillin, oxacillin, erythromycin and polymyxin B) to reduce their minimum inhibitory concentration against *K. pneumoniae* (up to 1000-fold reduction).^23^

The most common coatings for catheters to inhibit bacterial attachment are based on a combination of chlorhexidine and silver sulfadiazine, with chlorhexidine associated with anaphylactic shocks.^24,25^ Organisms such as *Acinetobacter baumannii* exhibit resistance to Chlorhexidine *via* overexpression of a chlorhexidine efflux protein.^26^ Moreover, discovery of resistance to silver,^27^ including complete resistance to nine different commercially available silver-based wound dressings, in *Klebsiella pneumoniae* and *Enterobacter cloacae* highlights the need for new coating strategies.

**Fig. 1:**
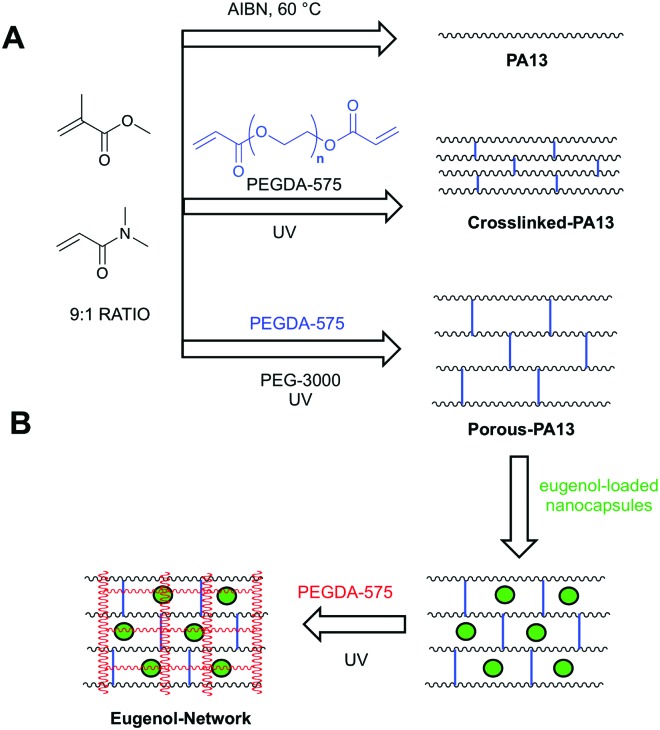
(A) Polymers based on methyl methacrylate and dimethylacrylamide (9 : 1 molar ratio) used in this study. PA13 was prepared by free radial polymerisation using azo*bisiso*butyronitrile (AIBN).^12^ Crosslinked-PA13 was prepared by photopolymerisation of PA13 monomers and 4.1% poly(ethylene glycol) diacrylate (PEGDA-575) as crosslinker. Porous-PA13 was prepared as above but with poly(ethylene glycol) (PEG-3000) included as a porogen. (B) Preparation of the fortified interpenetrating network. Porous-PA13 (first network) was incubated with eugenol containing poly(lauryl acrylate) nanocapsules followed by the entrapment of the nanocapsules by the second network composed of PEGDA-575.

Here, eugenol was investigated as antibacterial agent for bacterial repellent coating. However, like chlorhexidine^28^ and silver sulfadiazine,^29^ eugenol is known to be toxic to mammalian cells^30^ at high concentrations (∼4% hemolysis at 2 mg mL^–1^).^19^ Therefore, a coating consisting of a bacteria repellent polymer PA13 with the slow-release of eugenol was envisaged. Poly(lauryl acrylate)-based nanocapsules encapsulating clove oil or eugenol were prepared and evaluated for their ability to inhibit growth of two of the most common hospital pathogens, methicillin resistant *S. aureus* (MRSA) and *K. pneumoniae*, on polymer surfaces (Table 1). A strategy to incorporate the nanocapsules into PA13 with slow-release of eugenol was investigated, resulting in the development of a ‘fortified interpenetrating polymer network’ with eugenol nanocapsules entrapped within a Porous-PA13 (first network) and a poly(ethylene glycol) diacrylate (second network) (Fig. 1). This polymer network was applied to two medical devices (a catheter and an endotracheal tube) with a luminescence based microbial viability assay and scanning electron microscopy (SEM) used to study binding of bacteria.

**Table 1:** Polymers and polymer networks used in this study (Fig. 1) (PDI = polydispersity index). For chemical structures see ESI Section 16

Polymer	Description
PA13	Co-polymer of methyl methacrylate and dimethylacrylamide (polymerised in a molar ratio of 9 : 1, *M*_w_ 411 kDa, PDI 3.4).^12^ Bacteria repelling polymer surface.
PA155	Co-polymer of hydroxyethyl methacrylate and dimethylaminoethyl methacrylate (polymerised in a molar ratio of 1 : 1, *M*_w_ 9.45 kDa, PDI 1.2).^12^ This bacteria-binding polymer^11^ was used as a control surface.
Crosslinked-PA13	PA13 crosslinked with 4.1 mol% poly(ethylene glycol) diacrylate (PEGDA-575).
Porous-PA13	Crosslinked-PA13 prepared with 3 kDa poly(ethylene glycol) as a porogen (∼17% w/w).
Eugenol-Network	Fortified interpenetrating polymer network prepared with Porous-PA13 as the first network and PEGDA-575 as the second network with eugenol nanocapsules entrapped in the polymer network.
Blank-Network	An interpenetrating network prepared with Porous-PA13 as the first network and PEGDA-575 as the second network with no nanocapsules.

## Materials and methods

Kolliphor® RH40 was obtained from BASF, HPLC grade water was from Fisher Scientific, eugenol (≥98%, natural) and all other chemicals were from Sigma. MRSA (ATCC 252) and *K. pneumoniae* (ATCC BAA1706) were purchased from the American Type Culture Collection. The UV light source used for all polymerisation experiments was a UVP (model CL-1000, 365 nm, 8 Watt, 1000 mJ cm^–2^). The UV light of the culture hood was used for sterilisation. PA13 and PA155 were synthesised as described previously.^12^

### Preparation of nanocapsules

**Fig. 2:**
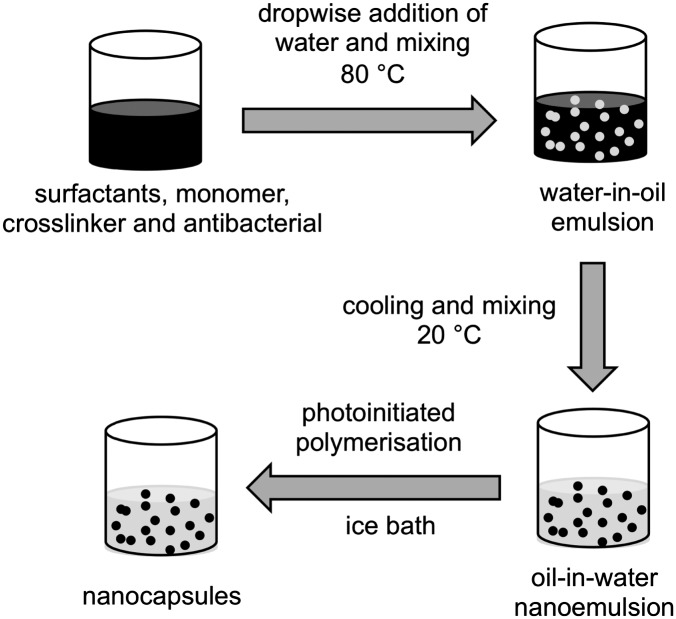
Synthesis of nanocapsules by phase inversion temperature method.

Nanocapsules were prepared by the phase inversion temperature method, as described previously^31^ with some modifications (Fig. 2). Nanoemulsions (5 mL scale) were prepared either as blank (no antibacterials), or with 5% of clove oil or eugenol. 0%, 1%, or 2% of crosslinker 1,6-hexanediol diacrylate, 10% lauryl acrylate, surfactants (7.5% Kolliphor® RH40 and 2.5% Span® 80) and eugenol (or clove oil) were heated to 80 °C in a glass vial and mixed vigorously using a magnetic stirrer until melting and homogeneous dissolution of all the components. Water (pre-heated to 80 °C) was added dropwise to the oil phase, the vial was sealed and the mixture stirred at 80 °C for 10 min to form a water-in-oil emulsion. The mixture was cooled to room temperature and mixed overnight to give an oil-in-water nanoemulsion. Where formulations formed viscous water-in-oil emulsions above their phase inversion temperature, they were either vortexed or shaken intermittently during the cooling stage. Irgacure® 2959 (1% w/w) was added to the nanoemulsions, mixed until complete dissolution, and the mixture placed in an ice bath before polymerisation under UV light for 1 h under continuous mixing. After irradiation, samples were mixed for 2 h at room temperature and stored under ambient conditions. Particle size distribution and polydispersity index was obtained by dynamic light scattering after each step (emulsification and polymerisation) using a Zetasizer (Nano-ZS, Malvern Instruments). The samples were diluted (10% v/v) with HPLC grade water with readings taken at 22 °C, with a scattering angle of 173°. See ESI,† Section 1 and Table S1 for composition optimisation studies.

### Transmission electron microscopy

Transmission Electron Microscopy (TEM) of the nanocapsules was performed using a Philips/FEI CM120 Biotwin Transmission Electron Microscope. Samples were diluted in water (5% v/v), dropped into a TEM grid holder and left to dry at room temperature overnight inside a desiccator prior to imaging.

### Antimicrobial assays

2× Mueller-Hinton broth was inoculated with 2 × 10^6^ CFU mL^–1^ MRSA or *K. pneumoniae* and challenged with equal volume of the nanocapsule dispersions (diluted in PBS to yield eugenol or clove oil concentrations of 0.625, 0.25, 0.1875, 0.125, and 0.025 mg mL^–1^) for 16 h allowing growth curves and IC_50_ values to be determined (ESI,† Section 10). Bacterial viability in nanocapsule-challenged media was compared with PBS (negative control) and hydrogen peroxide (8% w/w) (positive control) using the BacTiter-Glo^TM^ (Promega) microbial viability assay (ESI,† Section 11).

### Bacterial attachment on polymer coated coverslips

Glass coverslips (19 mm diameter) were coated with PA13, PA155 and Crosslinked-PA13 (ESI,† Sections 3 and 4). 400 μL of undiluted non-crosslinked nanocapsule dispersions were pipetted on to the coverslips in a 6-well plate and spread over the surface. After 1 h incubation, the coverslips were washed with PBS (2 × 2 mL) and sterilised by UV light for 30 min before inoculating with bacteria. Luria-Bertani (LB) broth inoculated with MRSA or *K. pneumoniae* (2 × 10^6^ CFU mL^–1^) was pipetted into the 6-well plates (2 mL per well) and incubated for 24 h at 37 °C. Media was removed and the coverslips washed with PBS (2 × 2 mL). The coverslips were incubated (1 h) with 10% formalin in PBS containing 1 μg mL^–1^ of Hoechst 33342, and washed with PBS (2 mL). Coverslips were imaged using the DAPI channel (*λ*_ex/em_ 357/447 nm) of an EVOS FL microscope with a 60× objective.

### Coating of medical devices

A central venous catheter (polyurethane, Arrow international, CS12123E) was cut into cylindrical pieces of approximately 5 mm in length (weight 41–45 mg) and an endotracheal tube (polyvinyl chloride, Rusch, 112482) was punched to obtain circular pieces of 10 mm diameter.

For coating with Porous-PA13, the surface of the catheter pieces was first roughened with a scalpel. Each piece was held at the central lumen and dip-coated with the polymerisation mixture containing the monomers, crosslinker, initiator, solvent and PEG-3000 (see ESI,† Section 5 for composition), followed by polymerisation under UV light for 1 h. The dip-coating and polymerisation was repeated holding the other side of the catheter piece. For coating the tracheal tube, roughened pieces were placed into a silicone mould (10 mm diameter, 5 mm depth) prepared using a silicone elastomer (Mold Star 15 Slow). 150 μL of the polymerisation mixture was pipetted onto the surface and photopolymerised for 1 h, and the process repeated on the other side. The catheter and tracheal tube pieces were washed thoroughly with water and incubated sequentially with 40 mL of water (2 × 15 min), 50% acetone in water (5 min) and water (15 min), followed by freezing (dry ice) and lyophilisation. The dried catheter and tracheal tube pieces coated with Porous-PA13 were soaked in the non-crosslinked eugenol containing nanocapsules (100 μL per sample) for 30 min, lyophilised for 2 h, and dipped into a poly(ethylene glycol) diacrylate (PEGDA, 575 Da) solution (containing PEGDA, Irgacure® 2959, methanol and water at 10%, 2%, 8% and 80% w/w, respectively) and photopolymerised for 30 min to give Eugenol-Network coating.

For coating with PA13 (control) the catheter and tracheal tube pieces were dipped into a 10% solution of PA13 in acetone, dried for 2 h under ambient conditions, coating repeated on the other side and the pieces dried overnight.^12^

### Reduction in bacterial binding on fortified interpenetrating polymer network

To quantify the reduction in bacterial binding by eugenol encapsulated nanocapsules trapped in an interpenetrating polymer network, monoliths of Porous-PA13, Eugenol-Network and Blank-Network were prepared (ESI,† Section 5 and 6) and quantification of surface-bound bacteria was performed using the process described previously.^32^ The monoliths (3 × 10 mm cylinders, *n* = 3) were sterilised under UV light (20 min each side), washed with water (2 × 40 mL), and placed in a 24 well-plate. MRSA and *K. pneumoniae* in LB broth (2 × 10^6^ CFU mL^–1^) were added (2 mL per well) (ESI,† Section 9) and incubated for 24 h at 37 °C. The monoliths were transferred to a clean 24-well plate, washed with PBS (2 × 2 mL), transferred to a 24-well plate containing LB broth (1 mL per well) and the media pipetted up and down 10 times vigorously to dislodge surface bound bacteria. Resultant cultures were incubated for 2 h at 37 °C (wells with 1 mL of media without bacteria used as control). 500 μL from each well was removed and added to 500 μL of BacTiter-Glo^TM^ reagent (prepared as per manufacturer's instructions) and mixed thoroughly by pipetting. 100 μL from each well was transferred to an opaque 96-well plate and luminescence was recorded using a Biotek Synergy HT well plate reader (autogain, shaking 5 min).

The surface-adhered bacteria on uncoated endotracheal tube and on monoliths of the Eugenol-Network and the Blank-Network (ESI,† Section 6) were determined by the same method.

### Scanning electron microscopy (SEM)

Catheter and endotracheal tube pieces, uncoated and coated with PA13 or Eugenol-Network, were sterilised with UV light (20 min each side) and incubated with the bacterial cocktail described above. After washing with PBS (2 × 2 mL), surface-attached bacteria were fixed with 10% formalin (2 mL per well, 1 h), washed with PBS, and dried in a fume hood overnight. The pieces were coated with a gold/palladium (60/40%) alloy using an Emscope SC500A sputter coater and SEM images obtained using a Hitachi 4700 II, cold field-emission Scanning Electron Microscope.

## Results and discussion

### Preparation of nanoemulsions and nanocapsules

Poly(lauryl acrylate) nanocapsules^31^ were prepared by photopolymerisation of oil-in-water nanoemulsions composed of an oil phase containing lauryl acrylate (10% w/w), eugenol or clove oil (5% w/w) and varying degrees of 1,6-hexanediol diacrylate (0, 1, 2% w/w) as a crosslinker (Fig. 2, ESI,† Table S1). Lauryl acrylate was chosen as the hydrophobic monomer due to its previous success in the efficient production of polymeric nanoparticles.^31^ Nanoemulsions were prepared by phase inversion temperature emulsification, which is a low energy technique utilising the temperature-dependent reduction in interfacial tension between aqueous and oil phases that is induced by non-ionic surfactants that incorporate polyethylene oxide.^33,34^ The reduced interfacial tension allows for better droplet break up and hence smaller particle sizes.^35^ Emulsification was conducted at 80 °C and the composition of the surfactants was optimised (7.5% Kolliphor® RH40 and 2.5% Span® 80) to achieve a stable nanoscale emulsion (ESI,† Section 1).

The nanoemulsion oil droplets containing the monomers and antimicrobials can be likened to ‘nanoreactors’^31^ undergoing polymerisation to form nanocapsules. TEM images of the nanocapsules prepared by *in situ* photopolymerisation of the nanoemulsions showed differing morphologies for the unloaded and eugenol or clove oil loaded nanocapsules (Fig. S2, ESI†). All nanocapsules had a size of <100 nm with a PDI of <0.16 (Fig. S3, ESI†).

### Antibacterial activity of eugenol and clove oil nanocapsules

Nanocapsules loaded with eugenol or clove oil with varying degree of crosslinking were assessed for their ability to inhibit growth of MRSA and *K. pneumoniae*. Both species were affected by the antibacterial loaded nanocapsules in a dose dependent manner (ESI,† Section 10), with >50% inhibition at a concentration of 0.625 mg mL^–1^ of eugenol or clove oil. *K. pneumoniae* showed greater susceptibility than MRSA with eugenol loaded nanocapsules showing ∼3-fold greater inhibition than clove oil. IC_50_ values for eugenol in nanocapsules (after 16 h incubation) for both bacteria were ≤0.35 mg mL^–1^, similar to free eugenol.^19^ The degree of crosslinking of the capsules did not influence their antibacterial activity. Evaluation of bacterial viability *via* BacTiter-Glo^TM^ microbial viability assay (ESI,† Section 11) confirmed the anti-bacterial activity of the eugenol and clove oil containing nanocapsules.

**Fig. 3:**
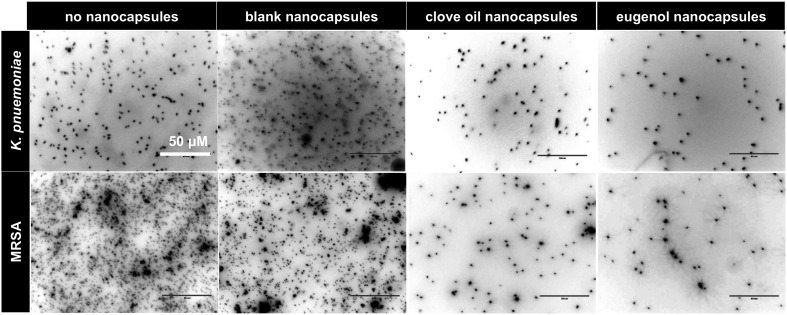
Coverslips coated with PA13 showing reduced binding of *K. pneumoniae* and MRSA for surfaces loaded with clove oil or eugenol containing nanocapsules (no crosslinker) when compared with blank-treated or untreated controls (scale bar 50 μm). PA13 coated coverslips treated with non-crosslinked nanocapsules were incubated with bacteria for 24 h, and the bacteria fixed and stained with Hoechst 33342. The coverslips were imaged in the DAPI channel (*λ*_ex/em_ 357/447 nm, 60× objective) and the images processed using ImageJ^TM^, providing an image of black bacteria against a white background.

To assess the ability of surface-coated nanocapsules to reduce bacterial binding, PA155 (a polymer that promotes bacterial binding^11^) coated coverslips were treated with nanocapsules, incubated with MRSA and *K. pneumoniae* for 24 h and the bacteria were fixed and stained. Analysis by fluorescence imaging after Hoechst 33342 staining showed a clear reduction of binding of both species on surfaces with the nanocapsules when compared to untreated controls (Fig. S11, ESI†). Coverslips coated with bacteria-repelling polymers PA13 and Crosslinked-PA13 also showed that addition of surface-coated nanocapsules further reduced bacterial binding on these polymers (Fig. 3 and Fig. S11, S12, ESI†) with eugenol encapsulated nanocapsules producing the best bacteria repelling surfaces (∼78–100% reduction of bacteria binding observed on PA13, PA155 and Crosslinked-PA13).

### Preparation of eugenol releasing fortified interpenetrating polymer network

Fortified interpenetrating polymer networks, encapsulating the eugenol-loaded nanocapsules, were prepared (ESI,† Section 6). This double network structure was required, as PA13 itself could not be used to trap nanocapsules (PA13 is insoluble in solvents compatible with the nanocapsules and *in situ* trapping during polymerisation was not possible due to incompatibly of the monomers with the nanocapsules) while Crosslinked-PA13 did not allow nanocapsule penetration (ESI,† Section 8.1).

**Fig. 4:**
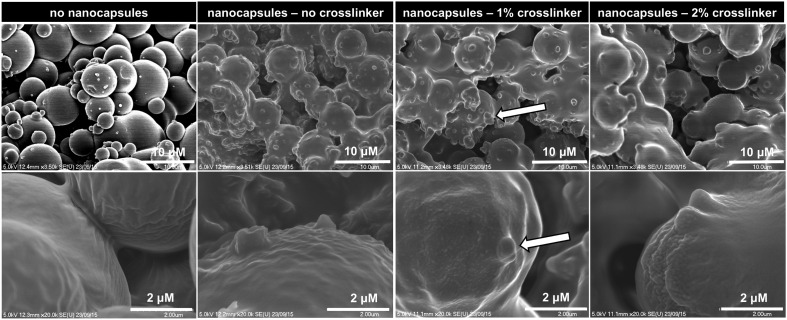
SEM images of sliced sections of Porous-PA13 monoliths incubated with blank nanocapsules with 0–2% crosslinker showed penetration of the nanocapsules (arrows) into the polymer network. Scale bar top row 10 μm (2000×) and bottom row 2 μm (3500×).

Firstly, monoliths of Porous-PA13 were prepared by photopolymerisation of monomers (methyl methacrylate and dimethylacrylamide in a 9 : 1 molar ratio) with the crosslinker poly(ethylene glycol) diacrylate (4.1 mol% of PEGDA-575) in MeOH^36^ with polyethylene glycol (3 kDa) as a porogen (ESI,† Section 5), which allowed incorporation of the nanocapsules into the polymer (Fig. 4, ESI,† Section 8.2).

For the preparation of the fortified interpenetrating polymer network, Porous-PA13 was incubated with non-crosslinked eugenol containing nanocapsules, which were subsequently trapped into the coating by introduction of a second polymer network prepared from poly(ethylene glycol) diacrylate (PEGDA-575) (ESI,† Section 6). This second network eliminates leaching of the nanocapsules (monoliths of Porous-PA13 without second network showed 60% nanocapsule release after 20 h incubation in water. See ESI,† Table S2). The Eugenol-Network had a nanocapsule content of 42 ± 3 μg mm^–3^ (calculated based on eugenol content per monolith, see ESI,† Section 7).

**Fig. 5:**
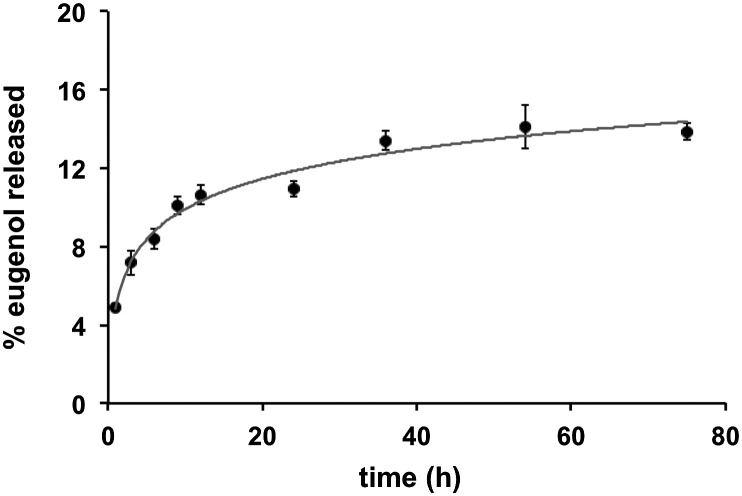
The time-dependent release of eugenol from the polymer coating. Monoliths of Eugenol-Network were incubated with 1 mL of water at room temperature and analysed by HPLC. 100% release was quantified by soaking the monoliths in 2 mL of methanol overnight followed by crushing and sonication and HPLC analysis.

Quantification of eugenol release from the Eugenol-Network *via* HPLC analysis showed a slow and controlled release with less than 10% of eugenol released after 24 h (15% after 72 h) (Fig. 5), suggesting that the coating can retain its anti-fouling nature for an extended duration. This is vital as catheters can remain in use for long periods, while burst release can lead to toxicity.

### Fortified interpenetrating polymer network reduces bacterial attachment and is biocompatible

**Fig. 6:**
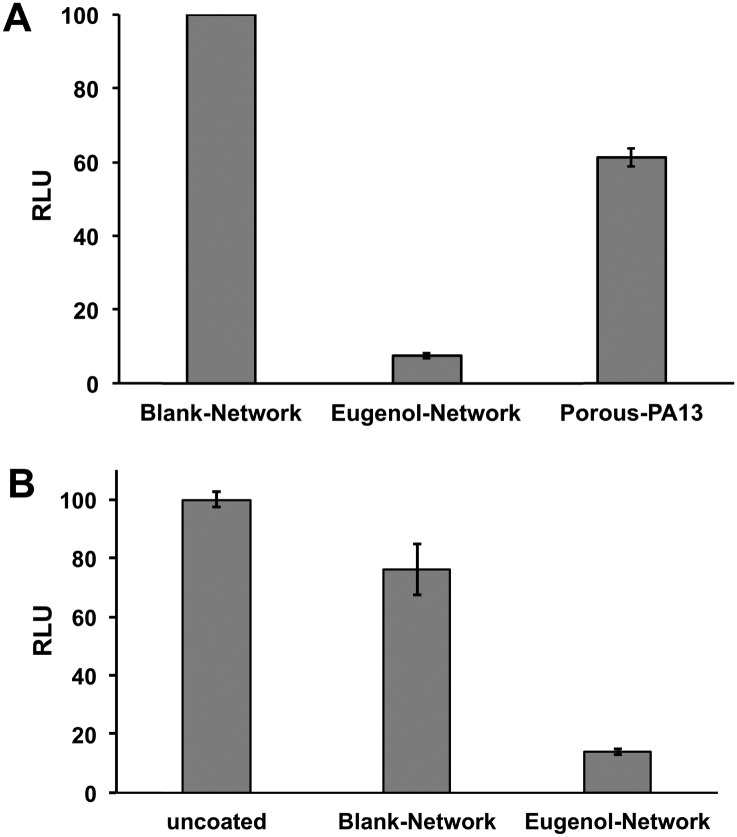
(A) Normalised relative luminescence intensity of media containing surface-bound MRSA and *K. pneumoniae* from monoliths of the Blank-Network, the Eugenol-Network, and Porous-PA13 (*n* = 3). The monoliths were incubated 24 h with the bacteria, washed, and surface bound bacteria detached and suspended in fresh media prior to BacTiter-Glo™ assay. (B) Normalised luminescence BacTiter-Glo™ assay of surface-bound MRSA and *K. pneumoniae* on the surface of uncoated endotracheal tube and on the Blank-Network and the Eugenol-Network monoliths (*n* = 3, with 3 × 10 mm pieces used for each assay).

This Eugenol-Network coating (prepared as a monolith) was assessed for its ability to inhibit binding of MRSA and *K. pneumoniae*. After overnight incubation with a cocktail of *K. pneumoniae* and MRSA, an ATP luminescence assay was used to quantify the level of surface-bound bacteria. The eugenol containing monoliths showed up to 90% reduction in luminescence compared to controls (Porous-PA13 and Blank-Network) (Fig. 6A), confirming the ability of the nanocapsules to reduce bacterial viability. Bacterial binding was also compared with the Eugenol-Network and a surface of a polyvinyl chloride endotracheal tube with the Eugenol-Network monoliths showing 86% fewer bacteria than the endotracheal tubes (Fig. 6B).

No hemolysis was observed after 1 h incubation of Eugenol-Network monoliths with human erythrocytes (ESI,† Section 14), confirming the biocompatibility of the coatings.

### Bacterial binding on the surface of coated catheters and endotracheal tubes

**Fig. 7:**
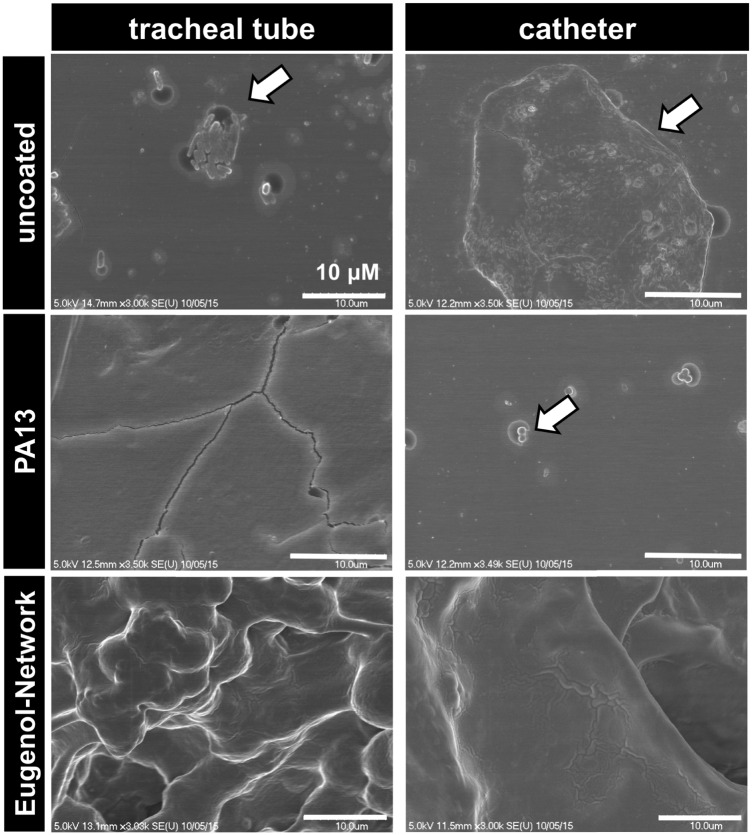
SEM images of uncoated and PA13 and Eugenol-Network coated endotracheal tube and catheter after incubation with a cocktail of *K. pneumoniae* and MRSA (scale bar 10 μm). The arrows show surface attached bacteria.

Two medical devices, a polyvinyl chloride based endotracheal tube and a polyurethane based central venous catheter, were coated with Porous-PA13, followed by incubation with eugenol containing nanocapsules, which were trapped by a second network. After incubation with a cocktail of *K. pneumoniae* and MRSA, surface bound bacteria were examined *via* SEM. The SEM images showed patches of bacterial colonisation on the uncoated medical devices, with no colonisation observed on the Eugenol-Network coating (Fig. 7). The observed reduction in bacterial binding, together with the biocompability and slow release of eugenol, clearly demonstrate the potential of these polymers for clinical applications.

## Conclusions

Poly(lauryl acrylate) based nanocapsules encapsulating eugenol were synthesised by the phase inversion temperature method and shown to inhibit growth and reduce binding of two major pathogens, Gram-negative *K. pneumoniae* and Gram-positive MRSA, demonstrating their potential for use in antibacterial coatings. The eugenol containing nanocapsules were successfully trapped within a porous polymer coating to give a ‘fortified interpenetrating polymer network’, which allowed slow release of eugenol without hemolytic activity. When applied as a coating on two medical devices this Eugenol-Network showed significant reduction in binding of bacteria showing the value of the coating as a means of combating medical-device associated infections.
