# Supplementary material for: Fortified interpenetrating polymers – bacteria resistant coatings for medical devices
Source: J Mater Chem B. 2016 Jul 18;4(32):5405–11. doi: 10.1039/c6tb01110a (PMC5038341; doi:10.1039/c6tb01110a)
Supplement: Supplementary file 1 [file TB-004-C6TB01110A-s001.pdf]

Electronic Supplementary Material (ESI) for Journal of Materials Chemistry B.
This journal is © The Royal Society of Chemistry 2016

Supplementary information

Fortified interpenetrating polymers – Bacteria resistant coatings for medical devices

Seshasailam Venkateswaran,[a,‡] Orlando David Henrique Dos Santos,[b‡] Emma Scholefield,[c] Annamaria Lilienkampf,[a] Peter J. Gwynne,[d] David G. Swann,[e] Kevin Dhaliwal,[c] Maurice P. Gallagher,[d*] and Mark Bradley.[a*]

[a] *EaStCHEM School of Chemistry, University of Edinburgh, King's Buildings, West Mains Road, Edinburgh, EH9 3JJ, UK. Email: mark.bradley@ed.ac.uk; Tel: +44 (0)131 650 4820*

[b] *Laboratório de Fitotecnologia, Departamento de Farmácia, Escola de Farmácia, Universidade Federal de Ouro Preto, Ouro Preto, Minas Gerais, 35400-000, Brazil*[c] *MRC Centre for Inflammation Research, The Queens Medical Research Institute, University of Edinburgh, 47 Little France Crescent, Edinburgh EH16 4TJ, UK.*

[d] *School of Biological Sciences, University of Edinburgh, King's Buildings, West Mains Road, Edinburgh , EH9 3JF, UK. Email: mp.gallagher@ed.ac.uk; Tel: +44 (0)131 650 5409*

[e] *Critical Care, NHS Lothian, Royal Infirmary of Edinburgh, 51 Little France Crescent, Edinburgh, EH16 4SA, UK*

[*]Corresponding authors, [‡]Equal contribution

Content

1. Preparation and characterisation of antibacterial nanoemulsions and nanocapsules S4
  1.1. Optimisation of surfactant composition for emulsification
  1.2. Composition and characterisation of nanoemulsions and nanocapsules
2. Preparation and characterisation of Dil nanocapsules S6
3. Preparation of **PA13** and **PA155** coated coverslips S7
4. Preparation of **Crosslinked-PA13** coated coverslips S7
5. Preparation of **Porous-PA13** monoliths S8
6. Preparation of monoliths of **Eugenol-Network** and **Blank-Network** S8
7. Quantification of eugenol release o from **Eugenol-Network** S8
  7.1. Construction of calibration curve
  7.2. Quantification of release of eugenol from **Eugenol-Network**.
  7.3. Quantification nanocapsules in **Eugenol-Network**
8. Optimisation of composition of **Crosslinked-PA13** and **Porous-PA13** S10
  8.1. Optimisation of molar ratio of monomers, crosslinker,and solvents
  8.2. Optimisation of porogen concentration for the preparation of **Porous-PA13**
  8.3. Effect of solvent composition on the migration of nanocapsules
9. Bacteria culture preparation S13
10. Bacterial growth inhibition S13
  10.1. Inhibition with nanocapsules
  10.2. Inhibition with **Eugenol-network**
11. Microbial viability assays S15
12. Influence of nanocapsules on bacterial attachment on **PA155** and **Crosslinked-PA13** S17
13. Quantification of bacterial attachment on polymer coated coverslips S18
14. Hemolytic activity of **Eugenol-Network** S18
15. Hemolytic activity of the nanocapsules S19
16. Chemical structures S20

Figures and Tables

Fig. S1 Optimisation of surfactant composition for emulsification.

Fig. S2 TEM images of nanocapsules.

Fig. S3 Particle size and polydispersity index of the nanoemulsions and nanocapsules.

Fig. S4 Flow cytometry histograms of fluorescent Dil nanocapsules.

Fig. S5 HPLC calibration for eugenol release.

Fig. S6 Retention of Dil nanocapsules in Crosslinked-PA13 and Porous-PA13 prepared with various compositions of crosslinker and solvents.

Fig. S7 Penetration of Dil nanocapsules into **Porous-PA13** monoliths prepared with NMP or MeOH.

Fig. S8 Growth curves of *K. pneumoniae* and MRSA treated with nanocapsules.

Fig. S9 IC_{50} values and % growth of MRSA and *K. pneumoniae* vs concentration of eugenol or clove oil in the nanocapsules.

Fig. S10 Screening of the nanocapsules for their antibacterial activity.

Fig. S11 Images showing the effect of nanocapsules on bacterial binding on **PA155** and **Crosslinked-PA13**

Fig. S12 Quantification of reduction in bacterial binding by nanocapsules on **PA13, PA155** and **Crosslinked-PA13**.

Table S1 Composition of the nanoemulsions as % weight (pre-polymerisation).

Table S2 Relative fluorescence intensity of the supernatant after 20, 30 and 60 h incubation of monoliths (with fluorescent nanocapsules) in water.

Table S3 Hemolytic activity of the **Eugenol-network** coating

Table S4 Haemolytic activity of the nanocapsules

1. Preparation and characterisation of antibacterial nanoemulsions and nanocapsules

1.1. Optimisation of surfactant composition for emulsification

Nanoemulsions containing 5% clove oil and 10% lauryl acrylate were prepared with HPLC grade water using the phase inversion temperature (PIT) method. The total concentration of the surfactant was maintained at 10% and various ratios (1:0, 3:1, 1:1, 1:3 and 0:1) of Span® 80 and Kolliphor® RH40 (hydrophilic–lipophilic balance values of 4 and 12 to 14, respectively) were explored to determine the surfactant composition that achieved stable nanoscale emulsions. Emulsification was conducted at 80 ± 2 °C for 10 min using an oil bath and mixing with a magnetic stirrer to form a water-in-oil emulsion. The samples were removed from the oil bath and cooled to below their PIT, with continuous mixing to room temperature (20 ± 2 °C), to obtain an oil-in-water emulsion. Ratios 3:1 and 1:0 of Kolliphor® RH40 and Span® 80, respectively, resulted in homogeneous emulsions (Fig. S1).

The droplet size and polydispersity index (PDI) of emulsions obtained with these two ratios were determined (Zetasizer Nano-ZS, Malvern Instruments) by diluting with HPLC grade water (10% v/v) and readings were taken at 22 °C, with a scattering angle of 173° using polystyrene disposable cuvettes. Kolliphor®RH40 and Span®80 at 3:1 ratio gave smaller and more defined particle size (96 nm, PDI 0.20) than 1:1 ratio (130 nm, PDI 0.24) and therefore was selected for emulsion preparations.

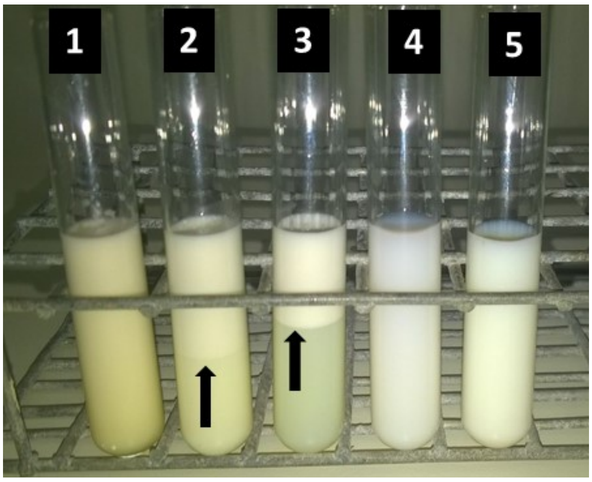

Fig. S1 Oil-in-water emulsions (containing 5% clove oil and 10% lauryl acrylate) prepared with various ratios of Kolliphor® RH40 and Span® 80 (total concentration 10%). Samples 1, 2 and 3 prepared with 0:1, 1:3 and 1:1 ratio of Kolliphor® RH40 and Span® 80, respectively, showed high viscosity (sample 1) or phase separation (samples 2 and 3, arrows indicating the interphase). Samples 4 and 5 prepared with 3:1 and 1:0 of Kolliphor® RH40 and Span® 80 showed no phase separation or increased viscosity.

1.2. Composition and Characterisation of nanoemulsions and nanocapsules

Table S1. Composition of the nanoemulsions as % weight (pre-polymerisation).

Formulation	Clove oil	Eugenol	Lauryl Acrylate	1,6-Hexanediol diacrylate	Kolliphor® RH40	Span® 80	Water
**Blank - No crosslinker**	0	0	10	0	7.5	2.5	80
**Blank - 1% crosslinker**	0	0	10	1	7.5	2.5	79
**Blank - 2% crosslinker**	0	0	10	2	7.5	2.5	78
**Clove oil- No crosslinker**	5	0	10	0	7.5	2.5	75
**Clove oil- 1% crosslinker**	5	0	10	1	7.5	2.5	74
**Clove oil- 2% crosslinker**	5	0	10	2	7.5	2.5	73
**Eugenol - No crosslinker**	0	5	10	0	7.5	2.5	75
**Eugenol - 1% crosslinker**	0	5	10	1	7.5	2.5	74
**Eugenol - 2% crosslinker**	0	5	10	2	7.5	2.5	73

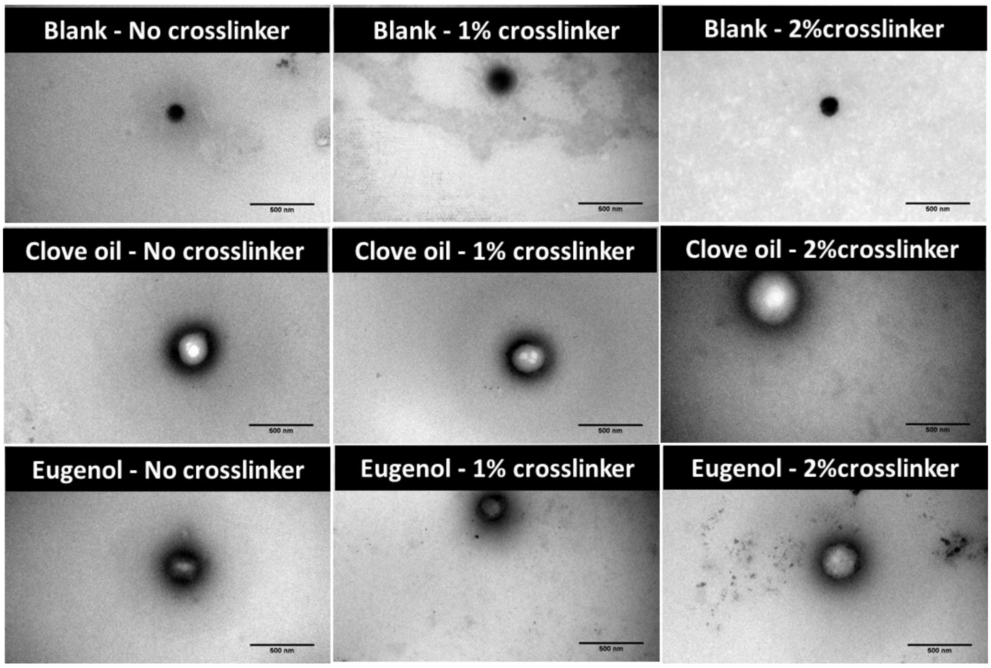

Fig. S2 TEM images (Scale bar = 500 nm) of the nanocapsules showed that the eugenol and clove oil containing nanocapsules had a core-shell structure, indicating potential encapsulation of the antimicrobials. The blank nanocapsules did not have a distinct core and shell.

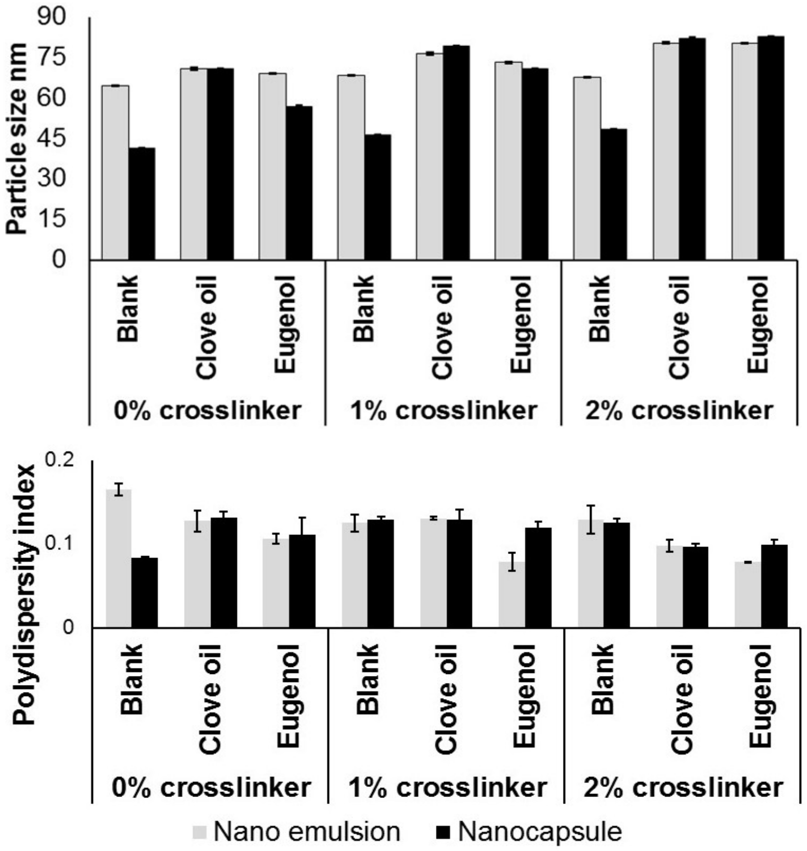

Fig. S3 Particle size and polydispersity index of the nanoemulsions and nanocapsules.

2. Preparation and characterisation of Dil nanocapsules

1,1'-Dioctadecyl-3,3,3',3'-tetramethylindocarbocyanine perchlorate (Dil) nanocapsules were prepared using the PIT method. Oil-in-water nanoemulsions were first prepared with Dil (0.06% w/w), lauryl acrylate (10% w/w), Kolliphor® RH40 (7.5% w/w), Span® 80 (2.5% w/w), and water (80% w/w). Polymerisation of nanoemulsions was conducted overnight in sealed glass vials in a fan-assisted oven at 40 ^{o}C, using ammonium persulphate (1% w/w) as the initiator and tetramethylethylenediamine (1% w/w) as the accelerator to produce Dil nanocapsules with particle size of < 100 nm and PDI of 0.2. Incorporation of Dil into the nanocapsules was confirmed by flow cytometry (BD FACSAria flow cytometer and the data analysed with FlowJo™) (Fig. S4).

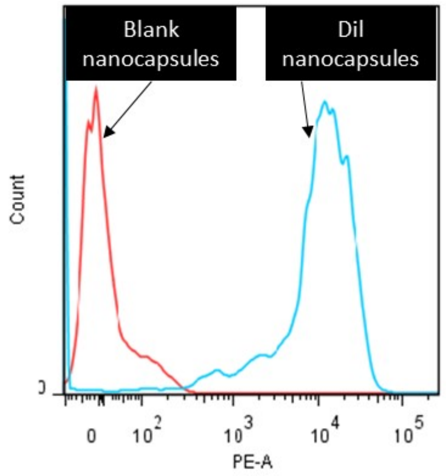

Fig. S4 Flow cytometry histograms showing the population shift of nanocapsules to higher fluorescence intensity when the Dil dye is incorporated in the formulation, confirming encapsulation of the dye.

3. Preparation of PA13 and PA155 coated coverslips

Polymers were spin-coated onto circular glass coverslips (19 mm diameter). 75 µL of 2% w/v polymer solutions in tetrahydrofuran were spin-coated at 2000 rpm for 10 s using a desktop spin coater (6708D, Speedline technologies). Coated coverslips were dried in a convection oven at 40 ºC overnight and sterilised for 30 min using UV light prior to inoculation.

4. Preparation of Crosslinked-PA13 coated coverslips

Prior to coating, the surface of glass coverslips (19 mm diameter) was functionalised with 3-(trimethoxysilyl)propyl methacrylate. Coverslips were placed one at a time in 10% NaOH (aq) in a glass beaker and agitated gently for 5 h. The coverslips were washed thoroughly with HPLC grade water and dried in an oven at 115 °C. The coverslips were transferred to a high density polyethylene container containing 30 mL acetonitrile, 3 mL trimethylamine and 6 mL with 3-(trimethoxysilyl)propyl methacrylate and agitated gently overnight at room temperature. The coverslips were washed with acetone (3 × 50 mL, swirled gently and decanted) and dried at 115 °C for 1 h in a glass container.

Methyl methacrylate and *N,N*-dimethylacrylamide were mixed in 9:1 molar ratio and 20% (w/w) 575 Da polyethylene glycol diacrylate (PEGDA-575) was added to give a monomer–crosslinker mixture. A polymerisation mixture was prepared with the monomer–crosslinker mixture (3.5 g, 70% w/w), Irgacure 2959 (0.1 g, 2% w/w), MeOH 0.9 g, 18% w/w) and water (0.5 g, 10% w/w) (for the optimisation of the composition see section 8.1). This mixture was pipetted (8 µL spots) on to acetate sheets and the 3-(trimethoxysilyl)propyl methacrylate functionalised coverslips were

placed gently over each spot. The acetate sheet was transferred to a UV source (CL-1000 Ultraviolet Crosslinker-UVP) and irradiated (UV-365 nm, 8 Watt, energy 1000 mJ cm^{-2}) for 90 min. The acetate sheets were dried overnight under ambient conditions. The coverslips were gently removed from the acetate sheets and immersed in excess water for 30 min and dried at 60 ^{o}C for 2 h in a fan-assisted oven. UV sterilisation (30 min) of coverslips was performed prior to inoculation.

5. Preparation of Porous-PA13 monoliths

Porous crosslinked polymer monoliths were prepared using 3 kDa polyethylene glycol (PEG-3000) as porogen. The polymerisation mixture (see section 4) was mixed with PEG-3000 (16.7% w/w). 200 µL of this solution was pipetted into cylindrical silicone moulds (diameter 10 mm and height 3 mm) and photopolymerised (1 h UV exposure). The polymer monoliths were removed from the moulds, soaked in acetone overnight (40 mL, gentle shaking), washed with acetone (× 2), soaked in acetone (50% v/v water, >3 h), followed by washing (× 2, acetone) and soaking in 40 mL of acetone (2 × 1 h). Monoliths were then dried *in vacuo* at room temperature.

6. Preparation of monoliths of Eugenol-Network and Blank-Network

For the **Eugenol-Network**, **Porous-PA13** monoliths were prepared (see section 5) and placed in a petri dish. 100 µL of eugenol containing nanocapsules without crosslinker (**Eugenol-No crosslinker**) was pipetted on the top surface of the monoliths, incubated (15 min), frozen with dry ice (15 min) and lyophilised (2 h), followed by dipping in a 575 Da poly(ethylene glycol) diacrylate (PEGDA-575) solution (containing PEGDA, Irgacure® 2959, MeOH and water at 10%, 2%, 8% and 80% w/w, respectively) and photopolymerised for 60 min. The monoliths were inverted and their underside coated with PEGDA-575 coating solution and photopolymerised for 60 min.

Blank-Network, were prepared without nanocapsules, *i.e.*, **Porous-PA13**, was directly coated with PEGDA solution and photopolymerised.

7. Quantification of eugenol release from Eugenol-Network

7.1 Construction of calibration curve

Calibration curve was constructed by HPLC using 0.5, 0.05, 0.005, 0.0005 and 0.00005 mg/mL of eugenol (in 50% MeOH/H_2O) (Fig. S5). The HPLC method was optimised to obtain sharp peaks for eugenol. Method: From 5% to 95% MeOH in water (with 0.1% formic acid) in 6 min, 6 min

isocratic, 1 min to 5% MeOH and then 2 min isocratic (Flow rate: 1 mL/min; Agilent 1100 HPLC, column: Kinetex 5μm XB-C18 100a, 50 × 4.60 mm; detection at 282 nm). Injection volumes for 0.5, 0.05, 0.005, 0.0005 and 0.00005 mg/mL standards were 10 μL, 25 μL, 100 μL, 100 μL and 100 μL, respectively. The area under the peak per μL of sample injected was calculated and a calibration curve was constructed.

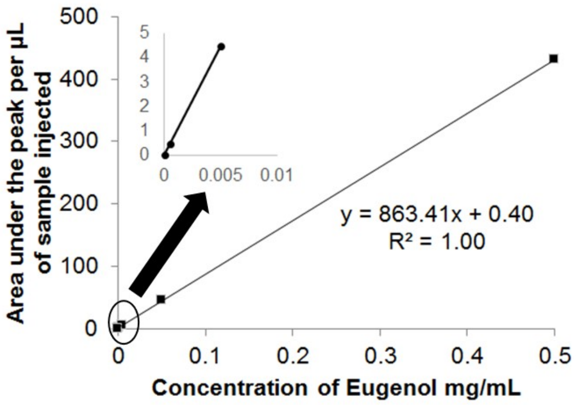

Fig. S5 HPLC (detection at 282 nm) calibration curve for eugenol (standards of 0.00005–0.5 mg/mL) prepared with a concentration range covering 4 orders of magnitude showed R^2 value of 0.99998. The inset shows the data points at 0.005, 0.0005 and 0.00005 mg/mL.

7.2 Quantification of release of eugenol from Eugenol-Network

Eugenol release from the eugenol encapsulating coating was quantified at various time points over a period of 3 days. Monoliths of **Eugenol-Network** (3 mm height and 10 mm diameter cylinders) were washed twice and incubated with 1 mL of HPLC grade water with gentle agitation (100 rpm, Edmund Buhler, KS15) (n = 3 per time point). Supernatant was removed (after pipetting up and down once) at every time-point (1, 3, 6, 9, 12, 24, 36, 54 and 75 h), mixed with equal volume of MeOH and analysed by HPLC (injection volume of 100 μL) to obtain the area under the curve per μL injection for eugenol peaks for every time point. % of eugenol release at each time-point was obtained by comparing the area under the curve per μL injection for eugenol peaks of each supernatant with that of the eugenol peak for 100% release.

To determine the total eugenol content of **Eugenol-Network** (*i.e.*, 100% release), monoliths of **Eugenol-Network** (n = 3) were soaked overnight in 2 mL of MeOH per monolith, followed by crushing and sonication. The supernatant was diluted 1:1 with water, further diluted 10 times by mixing with MeOH/water (1:1) and analysed by HPLC to be 3.3 mg eugenol per monolith. The nanocapsules had 50 mg/mL eugenol (based on nanocapsule preparation), therefore 65% of the total eugenol content was incorporated in the monoliths.

7.3 Quantification of nanocapsules in the Eugenol-Network

The total eugenol content was determined using HPLC (as detailed in ESI, section 7.1 and 7.2) to be 3.3 ± 0.2 mg (average ± SE, n = 3) of eugenol per monolith of **Eugenol-Network** (3 mm height and 10 mm diameter cylinders). Since the nanocapsule dispersions contain 15% nanocapsules (with 10% poly(laurylacrylate), encapsulating 5% eugenol), 9.9 ± 0.6 mg of nanocapsules were incorporated per monolith. Therefore the **Eugenol-Network** contained 42 ± 3 μg of the nanocapsules per mm^3 of the coating.

8. Optimisation of composition of Crosslinked-PA13 and Porous-PA13

8.1 Molar ratio of MMA:DMAA:PEGDA-575 and ratio of water

PA13 monomer mixture (in 9:1 molar ratio of MMA and DMAA) was mixed at various weight ratios with 575 Da poly(ethylene glycol) diacrylate (PEGDA-575,) to form monomer–crosslinker mixtures with 80%, 60%, 40% and 20% by weight of PEGDA-575. Each monomer–crosslinker mixture was mixed with Irgacure® 2959 (2% w/w in MeOH), 18% MeOH, and varying concentrations of water (0%, 10%, 20% or 30%). 8 μL spots (n = 3) of the polymerisation mixture were pipetted on to acetate sheets and a 3-(trimethoxysilyl)propyl methacrylate functionalised coverslip was placed over each spot and polymerised for 90 min using UV light. Coverslips were removed from the acetate sheets, placed in a 12-well plate and dried in an air assisted oven for 6 h (40 ^{o}C), washed and hydrated with HPLC grade water (4 h), excess water removed and dried (4 h at 40 ^{o}C). The coverslips were incubated with 75 μL of Dil nanocapsules (48 h at 2 ^{o}C) and washed with water (1 mL) to remove unbound nanocapsules.

Fluorescence intensity of coverslips was measured using a well plate reader (Biotek, λ_{Ex} 530/25; λ_{Ex} 590/35, with 5×5, equally spaced points analysed within the bottom of the well) followed by washing with water (4 × 1 mL). % of reduction in fluorescence was measured by comparing the fluorescence before and after 4 washes. The formulations with 20% crosslinker showed the least reduction in fluorescence (therefore the best retention of the nanocapsules), with the retention consistent for various concentrations of water in the polymerisation mix (Fig. S6). Visual examination of the polymerisation mixtures revealed that those with 0% and 10% water showed no phase separation. In order to obtain uniform polymerisation when used for coating, the optimised polymerisation mixture contained (w/w) 18% MeOH, 10% water, 2% initiator, and 70% monomer–crosslinker mixture (with 20% crosslinker). The molar ratio of MMA, DMAA and PEGDA-575 was 86.4: 9.4: 4.2, respectively.

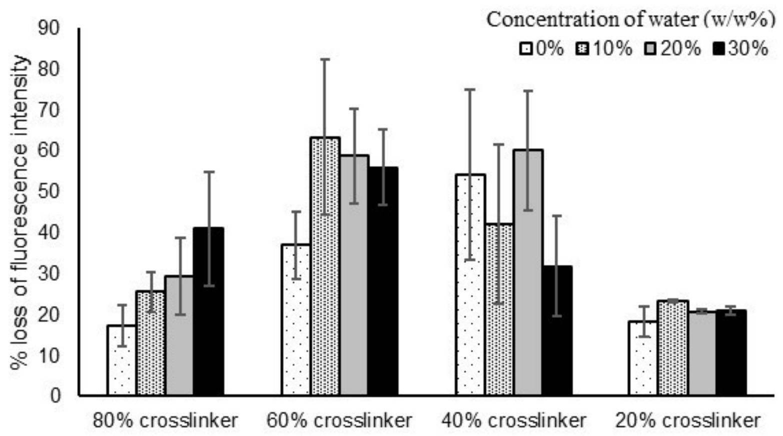

Fig. S6 Reduction of fluorescence intensity (%) of coverslips with various polymer compositions. The coverslips (n = 3) were treated with 75 μL of Dil nanocapsules, incubated for 48 h and washed with water. The best retention of nanocapsules was achieved with 20% of crosslinker.

8.2 Optimisation of porogen concentration for the preparation of Porous-PA13

The optimised polymerisation mixture (Section 8.1) was mixed with 0.0%, 2.0%, 4.8%, 9.1% or 16.7% w/w of 3 kDa poly(ethylene glycol) (PEG-3000). 200 μL of these solutions were pipetted into cylindrical silicone moulds (diameter 10 mm and height 3 mm) and polymerised with UV light (1 h). The polymer monoliths were then removed from the moulds, washed with acetone and acetone/water and dried (see section 5).

Each of the polymer monoliths were placed in a well of 24 well-plate and 100 μL of Dil nanocapsules was added over each monolith. After 15 minutes of incubation and visual inspection, only the formulation containing 16.7% PEG-3000 allowed penetration of the nanocapsules (with the entire monolith appearing uniformly coloured by pink Dil nanocapsules), while the Dil nanocapsules stayed on the surface of monoliths for all other PEG-3000 concentrations.

8.3 Effect of solvent composition on the migration of nanocapsules into porous-PA13 monoliths

MeOH and *N*-methyl-2-pyrrolidone (NMP) were investigated for their ability to induce pore formation during polymerisation to allow penetration of nanocapsules into the monoliths/coatings. **Porous-PA13** monoliths were prepared with MeOH and NMP as the solvent and their ability to permeate the nanocapsules was compared. After incubation (4 h) of the monoliths with 100 μL of Dil nanocapsules (in a 24 well-plate), only the monoliths prepared with MeOH allowed penetration of nanocapsules (the entire monolith appeared uniformly coloured on visual inspection), while the nanocapsules stayed on the surface on the NMP based monoliths. Next, the monoliths were

incubated 20 h in water (2 mL in a 24 well-plate with gentle shaking) and fluorescence of supernatant (200 µL) was measured in a 48 well-plate (Biotek plate reader, λ_{Ex} 530/25; λ_{Em} 590/35). The monoliths prepared with NMP showed no retention of nanocapsules after washing with water (2 mL) while those prepared with MeOH retained the nanocapsules within the matrix (Fig. S7). The washed monoliths were then incubated 10 h in water (2 mL) followed by fluorescence measurement of the supernatant. Fluorescence was measured again after another washing and incubation with water (2 mL, 30 h) (Table S2).

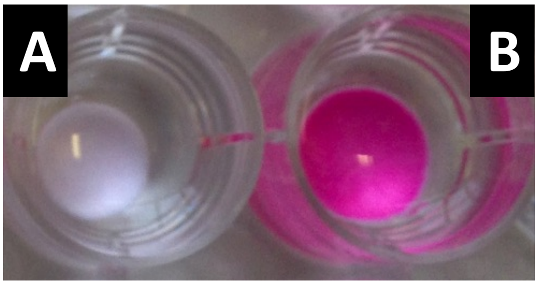

Fig. S7 Monoliths prepared with NMP (A) did not allow penetration of Dil nanocapsules (pink) with all the nanocapsules held on the surface and released, while the monolith prepared with MeOH (B) allowed penetration of nanocapsules and retained the colour of the Dil nanocapsules after incubation in water (20 h).

Table S2 Relative fluorescence intensity of the supernatant after 20, 30 and 60 h incubation of **Porous-PA13** monoliths (with Dil nanocapsules) in water confirmed the retention and continued release of the nanocapsules from the polymer matrix for the monoliths prepared with MeOH. Water and Dil nanocapsules (100 µL in 2 mL of water) were used as controls.

	**RFU 20 h**	**nanocapsules released**	**RFU 30 h**	**nanocapsules released**	**RFU 60 h**	**nanocapsules released**
**Monoliths prepared with NMP**	21346	100%	138	–	33	–
**Monoliths prepared with MeOH**	11912	60%	1462	7%	1372	7%
**Water**	18	–	18	–	20	–
**Dil nanocapsules**	20003	–	19811	–	19538	–

SEM was used to confirm penetration of nanocapsules with **Porous-PA13** monoliths prepared with MeOH. The monoliths were incubated (15 min) with 100 µL of nanocapsules **Blank-No Crosslinker**, **Blank-1% Crosslinker**, **Blank-2% Crosslinker**, frozen with dry ice (15 min) and dried *in vacuo* at room temperature (2 h). Sections of the monoliths with and without nanocapsules were coated with a Gold/Palladium (60%/40%) alloy using an Emscope SC500A sputter coater and imaged using a Hitachi 4700 II cold Field-emission Scanning Electron Microscope.

9. Bacteria culture and preparation

Overnight cultures of Methicillin resistant *Staphylococcus aureus* (MRSA) (ATCC252) and *Klebsiella pneumoniae* (*K. pneumoniae*) (ATCC BAA1706) were set up in LB broth (37 ^{o}C with shaking at 300 rpm, Incushake MIDI). 100 µL of the culture was added to 10 mL of fresh LB broth (in 50 mL falcon tubes) and incubated 2 h at 37 °C with shaking (300 rpm) to obtain a sub-culture of each strain. The sub-cultures were centrifuged (Megafuge 1.0, 3000 rpm for 10 min), supernatant removed, and 1 mL of PBS added. The suspension was mixed thoroughly by pipetting up and down, vortexed, transferred to a 2 mL eppendorf tube and (13000 rpm for 1 min, Sigma 1-13). Cells were washed with PBS (2 × 1 mL, vortexing and centrifugation at 13000 rpm for 1 min) and suspended in 1 mL of PBS. Optical density (OD) measurements were used to count the bacteria (assuming 1 OD = 10^9 bacteria) by measuring absorbance at 595 nm using a WPA, UV 1101 biotech photometer and polystyrene cuvettes (10×4×45 mm, Sarsted AG & Co.).

10. Bacterial growth inhibition

10.1 Inhibition with nanocapsules

Nanocapsules (containing 50 mg/mL of eugenol or clove oil) were diluted in PBS (25, 10, 7.5, 5 and 1 µL of nanocapsule solution per mL). 2X Mueller-Hinton broth with 2 × 10^6 CFU/mL of MRSA and *K. pneumoniae* was pipetted on a 96-well plate (50 µL per well) and 50 µL of the diluted nanocapsules (or 50 µL of PBS control) were added to each well, to give final concentrations of 0.625 0.25, 0.1875, 0.125, and 0.025 mg/mL of eugenol or clove oil. The well plate was sealed with an optically clear sealing film and absorbance was measured at 600 nm (Biotek Synergy HT plate reader, endpoint kinetic, every 15 min for 16 h at 35 °C, with 5 s shaking before every reading). Background absorbance at 0 min was deducted and growth curves were constructed (Fig. S8). % growth was calculated with the formula below by comparing the absorbance (after 16 h) of the nanocapsule-treated samples with PBS-treated samples.

$$\% \, growth = \frac{At - A0}{Bt - B0} X \, 100$$

A_t and A_0 are the absorbance of the nanocapsule-treated media at 16 h and 0 h, respectively; B_t and B_0 are the absorbance of the PBS-treated media at 16 h and 0 h, respectively. % growth was plotted against the concentration of eugenol or clove oil (in the total volume of the well) (Fig. S9). IC_{50} values were calculated by liner interpolation of these curves (Table S3).

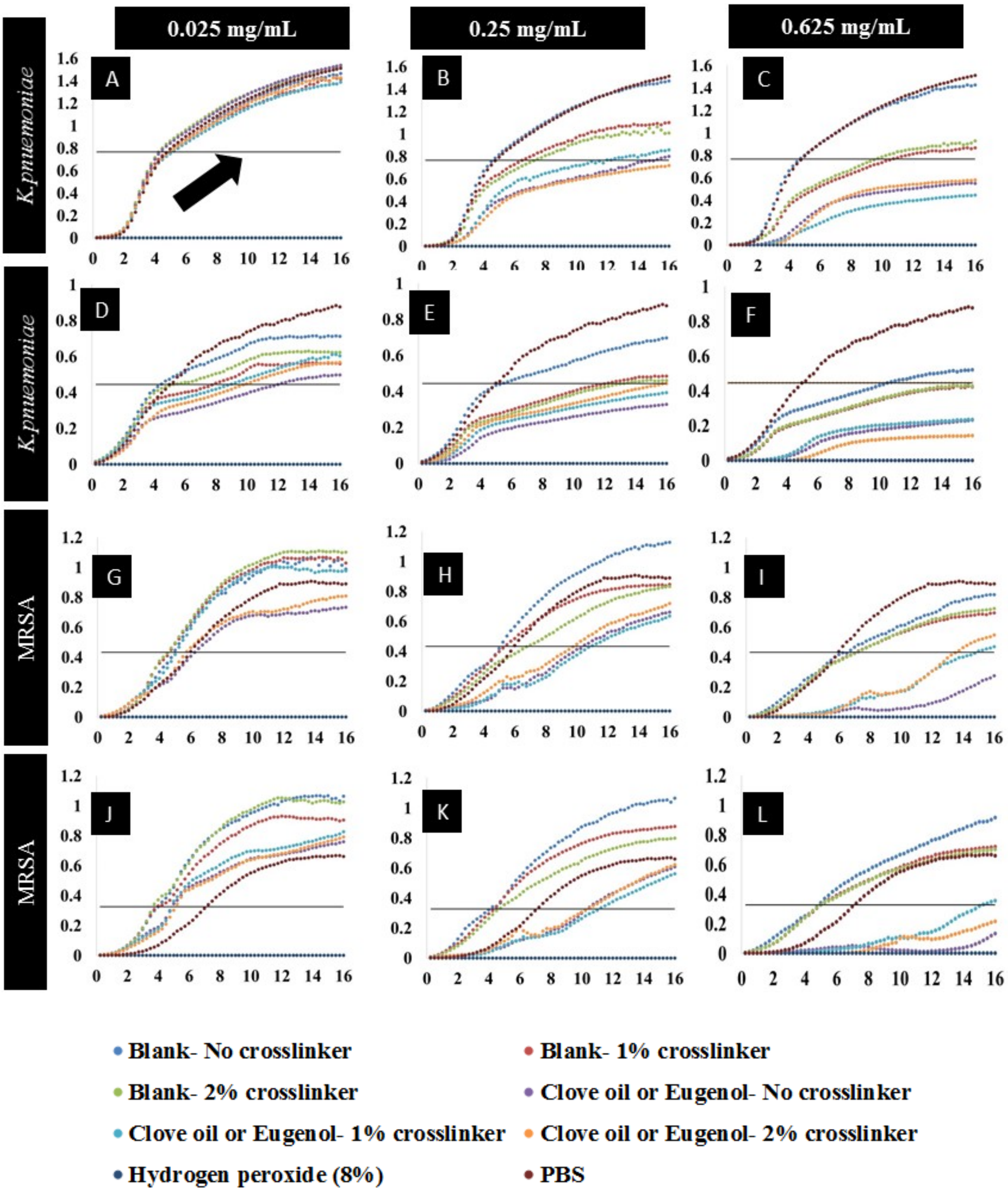

Fig. S8 Growth curves of *K. pneumoniae* and MRSA when challenged with various concentrations of nanocapsules (n = 3) resulting in 0.025, 0.25 and 0.625 mg/mL clove oil (A–C and G–I) or eugenol (D–F and J–L). Y-axis = absorbance at 600 nm, X-axis = time (h). The horizontal lines (shown by arrow in A) in each graph denote the 50% of the absorbance of corresponding PBS-treated media after 16 h.

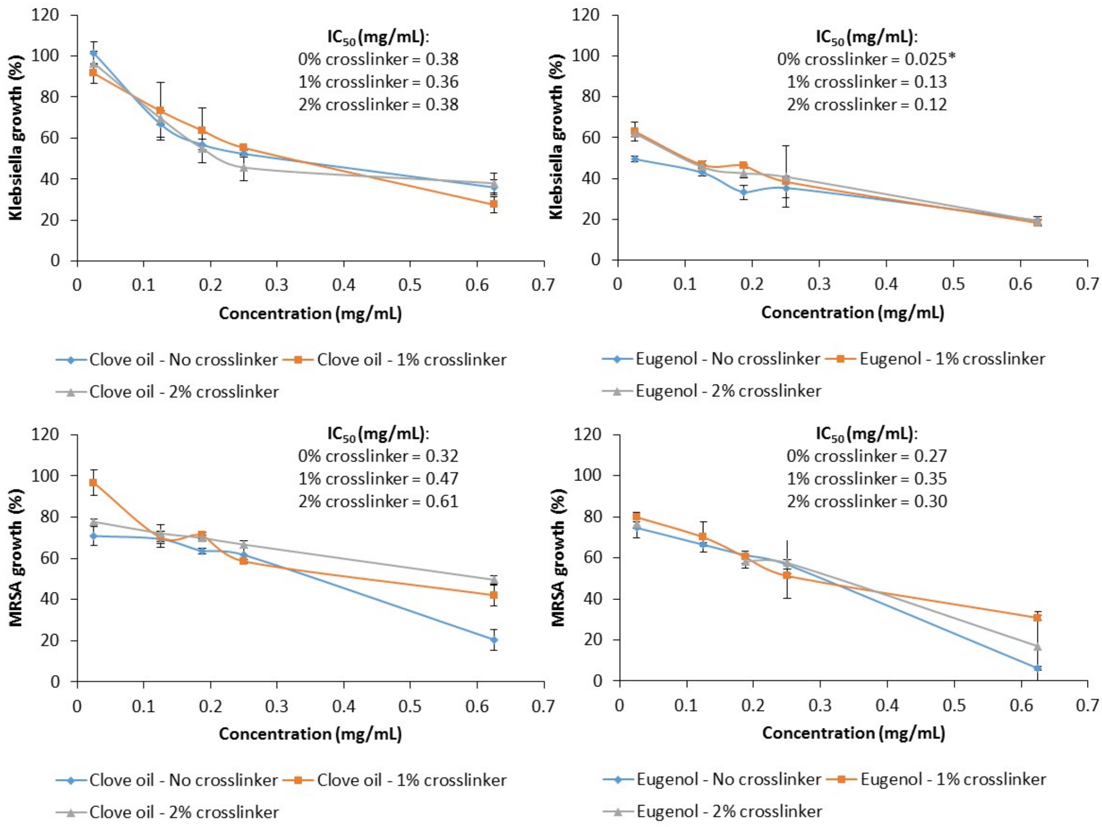

Fig. S9 % Growth of MRSA and *K. pneumoniae vs* concentration of eugenol or clove oil shows a dose-dependent reduction in growth. All IC_{50} values were generated by linear interpolation of curves, except for * where one of the samples tested (*i.e.*, 0.025 mg/mL) gave close to 50% reduction in growth.

10.2 Inhibition with Eugenol-network

% inhibition of growth of a cocktail of MRSA and *K. pneumoniae* was determined. The monoliths of **Eugenol-network** and **Blank-network** (n = 3) were sterilised under UV light (20 min each side), washed with water (2 × 40 mL), and placed in a 24 well-plate. MRSA and *K. pneumoniae* in LB broth (2×10^6 CFU/mL) were added (2 mL per well) (ESI, section 9) and incubated for 24 h at 37 °C. The monoliths were removed and the absorbance (600 nm) of the media measured. After subtracting the background absorbance (media without bacteria), the corrected absorbance of **Eugenol-network** and **Blank-network** were compared and % inhibition was determined to be 64 ± 3%.

11. Microbial viability assays

The antibacterial activity of nanocapsule dispersions was assessed using the BacTiter-Glo™ microbial viability assay (Promega). MRSA and *K. pneumoniae* in 2X Mueller-Hinton broth containing 2×10^6 CFU/mL was pipetted in a 96-well plate (50 µL per well) and 50 µL of the

diluted nanocapsules (50, 25 and 10 µL of nanocapsule dispersion per mL PBS) were added to each well, and the plates incubated at 35 °C for 1 h. PBS was used as a negative control and hydrogen peroxide (8% w/w) as a positive control. 50 µL from each well was transferred to an opaque 96-well plate and 50 µL of the BacTiter-Glo™ reagent (prepared as per manufacturer's instructions) was added. After sealing the well plate with an optically clear sealing film, luminescence was recorded (Biotek Synergy HT plate reader, gain = autogain, after shaking 5 min). Background luminescence was subtracted (media without bacteria) and the relative luminescence units (RLU) plotted against sample number (Fig. S10).

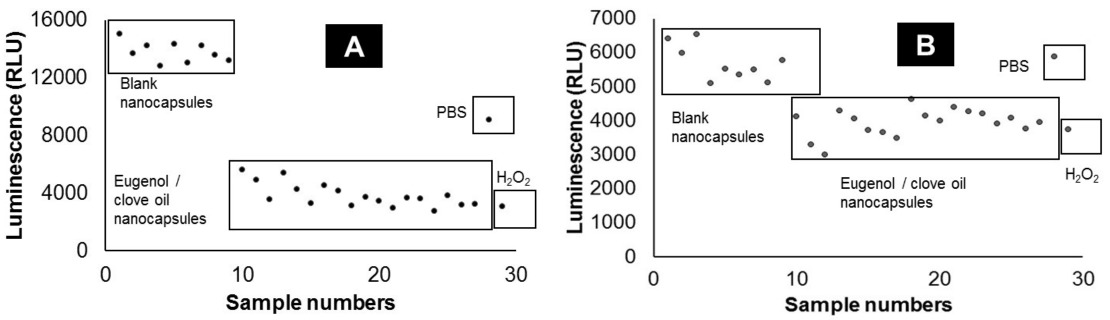

Fig. S10 Screening of the nanocapsules **Clove oil–No crosslinker**, **Eugenol–No crosslinker** and the control **Blank–No crosslinker** for their antibacterial activity using the BacTiter-Glo™ assay. The x-axis contains sample number and the y-axis is the average luminescence from bacteria after 1 hour incubation with the nanocapsules (n = 3). Three concentrations were tested for each type of nanocapsule (equivalent to 0.25, 0.625 and 1.25 mg/mL of eugenol or clove oil). Lower luminescence intensity was observed for bacteria treated with eugenol or clove oil containing nanocapsules (samples 10 to 27) than for blank nanocapsules (samples 1 to 9) and PBS (sample 28), and comparable luminescence to the positive control hydrogen peroxide (sample 29), indicating antimicrobial activity against both *K. pneumoniae* (A) and MRSA (B).

12. Influence of nanocapsules on bacterial attachment on PA155 and Crosslinked-PA13

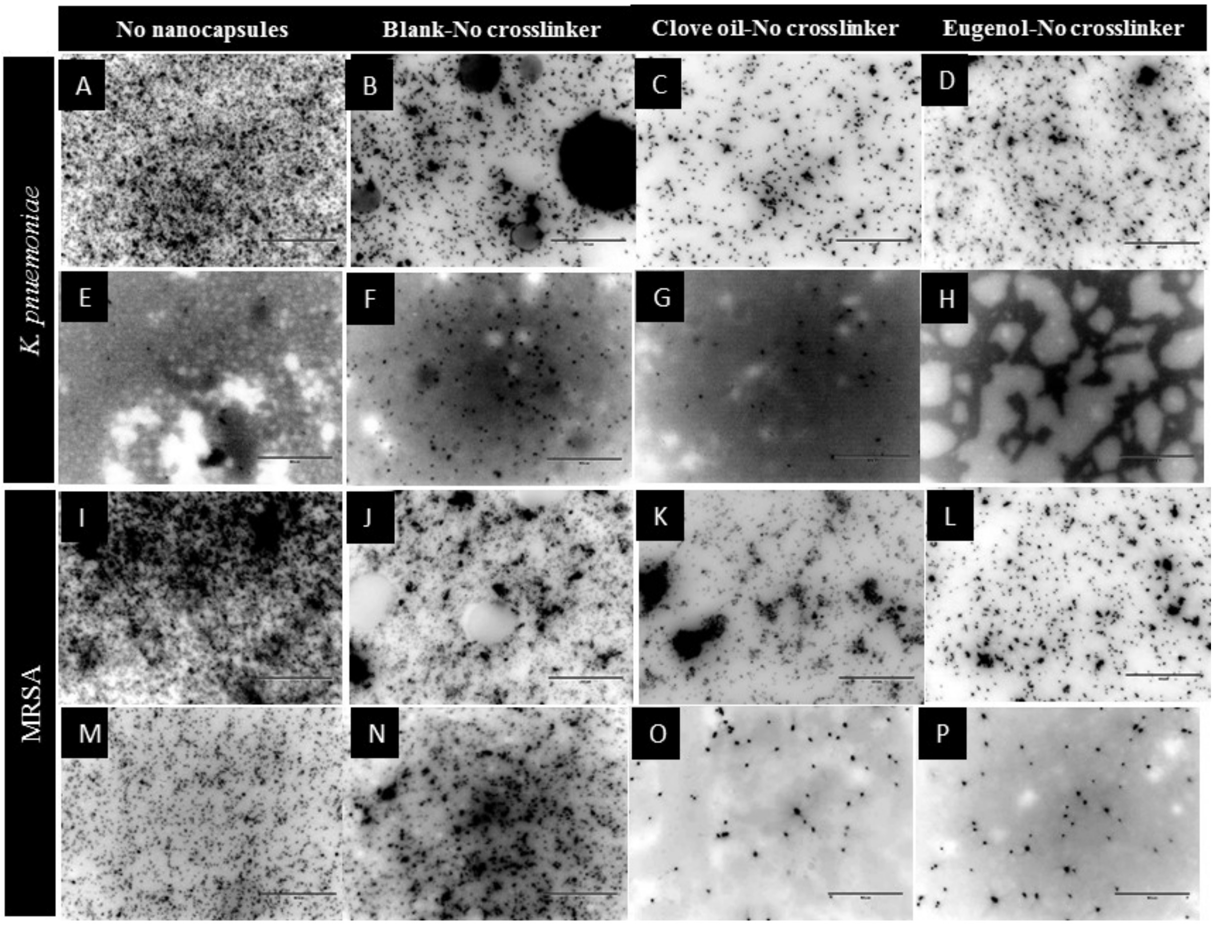

Fig. S11 Images (scale bar 50 µm) of coverslips coated with **PA155** (A–D and I–L) and **Crosslinked-PA13** (E–H and M–P) untreated or treated with nanocapsules **Blank–No crosslinker**, **Clove oil–No crosslinker** or **Eugenol–No crosslinker** show reduced binding of both *K. pneumoniae* and MRSA for coverslips treated with clove oil and eugenol containing nanocapsules. The coverslips were incubated with bacteria for 24 h, washed and the bacteria fixed and stained with Hoechst 33342. The coverslips were then imaged in the DAPI channel using EVOS FL microscope with a 60× objective. The images were processed using ImageJ using the *Split channels* function, followed by the *Invert* function, which provided a black image of the bacteria against a white background.

13. Quantification of bacterial attachment on polymer coated coverslips

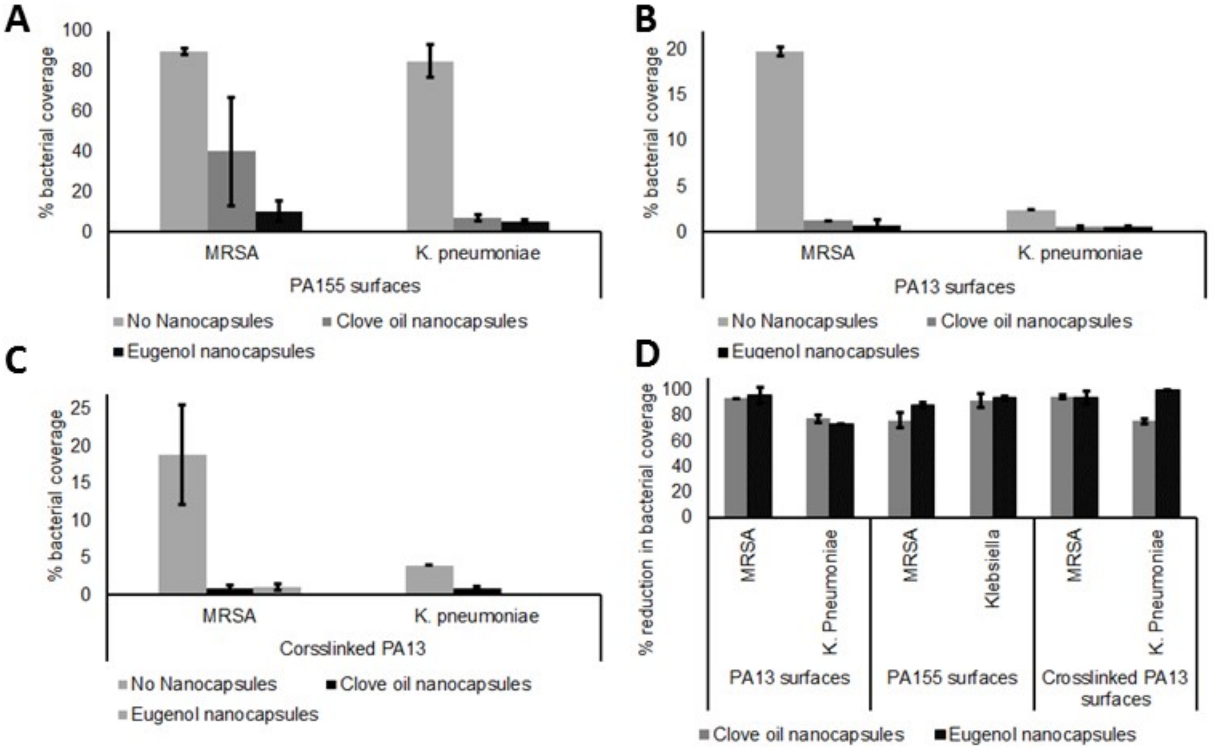

Fig. S12 Polymer coated coverslips, untreated or treated with eugenol or clove oil nanocapsules (no crosslinker) were incubated with MRSA or *K. pneumoniae* for 24 h, and the bacteria fixed and stained with Hoechst 33342. The coverslips were imaged in the DAPI channel ($\lambda_{ex/em}$ 357/447 nm, 60× objective) and the images processed using ImageJ^{TM}, providing an image of black bacteria against a white background (Fig. 3 and Fig. S11). The Percentage of bacterial coverage was determined from the images (n = 2) using ImageJ^{TM} (using *Threshold* and *Measure* functions). (A) **PA155** showed 90% surface coverage for both species. **PA155** with eugenol nanocapsules showed 10% and 5% coverage of MRSA and *K. pneumoniae,* respectively. (B) **PA13** showed 20% coverage of MRSA and 2% of *K. pneumoniae,* whereas **PA13** with eugenol nanocapsules showed 0.5% coverage of both species. (C) **Crosslinked-PA13** showed 19% coverage of MRSA and 4% of *K. pneumoniae,* whereas **Crosslinked-PA13** with eugenol nanocapsules showed 1% and 0% coverage. (D) Eugenol and clove oil nanocapsules produced a significant reduction in bacterial binding with eugenol nanocapsules providing a better performance.

14. Hemolytic activity of Eugenol-network

A suspension of human erythrocytes (obtained by centrifugal sedimentation of human whole blood from healthy donors (ethics approval from AMREC; 15-HV-013) was prepared (20% v/v in PBS). 4 mL of erythrocyte suspension was added to **Porous-PA13**, **Blank-Network** and **Eugenol-Network** in a 6-well plate and incubated at 37 °C (n = 3). PBS was used as negative control and Triton X-100 (4%) was used as the positive control. After 1 h incubation the samples were mixed thoroughly and 500 µL of erythrocyte suspension was placed into a 24-well plate containing 1 mL of PBS per well.

The well plates were sealed and centrifuged for 6 min at 2000 g. 100 µL of supernatant was removed from each well and hemolysis was determined by comparing the absorbance (540 nm, Biotek synergy HT plate reader) of the samples against the controls. All the polymer samples showed the same absorbance values as the negative control PBS, confirming no hemolysis (Table S3).

Table S3 Hemolytic assay with erythrocyte suspension (n = 3).

	**Absorbance (540 nm)**
**Porous-PA13**	0.04 ± 0.001
**Blank-Network**	0.04 ± 0.001
**Eugenol-Network**	0.04 ± 0.003
**PBS**	0.04 ± 0.001
**Triton X100 - 0.4%**	3.70 ± 0.258

15. Hemolytic activity of the nanocapsules

50 µL of erythrocyte suspension (20% v/v in PBS) and 50 µL of the nanocapsules (no crosslinker) in PBS were added to a 96-well plate, to give final concentrations of 0.625 0.25, 0.1875, 0.125, and 0.025 mg/mL of eugenol or clove oil and incubated at 37 °C (n = 3). PBS was used as negative and Triton X-100 (4%) was used as the positive control. After 1 h incubation, the samples were mixed thoroughly and 100 µL of PBS was added to each well. The well plates were sealed and centrifuged (5 min, 2000 g). 100 µL of supernatant was removed from each well and hemolysis was determined by comparing the absorbance (540 nm, Biotek synergy HT plate reader) of the samples against the controls. % hemolysis was calculated comparing the absorbance of sample against the absorbance of Triton X-100 (100 % hemolysis) and PBS (0% hemolysis). The eugenol nanocapsules showed higher hemolysis than the nanocapsules based on clove oil and the blank controls (no hemolysis) (Table S4).

Table S4. Hemolysis with nanocapsules at 25–625 µg/mL of eugenol. Erythrocyte suspension (20% v/v in PBS) was incubated h with eugenol nanocapsules (no crosslinker) followed by dilution with 100 µL of PBS. After centrifugation (5 min), absorbance (540 nm) of the supernatant was measured. Percentage of hemolysis was calculated by comparing the absorbance of sample against the absorbance of controls Triton X-100 (100 % hemolysis) and PBS (0% hemolysis).

Sample	25 µg/mL	125 µg/mL	188 µg/mL	625 µg/mL
**Blank- 0% crosslink**	-0.10 ± 0.13	-0.15 ± 0.02	-0.08 ± 0.04	0.05 ± 0.00
**Clove oil- 0% crosslink**	0.05 ± 0.08	3.09 ± 0.57	4.90 ± 0.69	12.66 ± 0.91
**Eugenol- 0% crosslink**	0.00 ± 0.00	2.22 ± 0.23	2.30 ± 0.35	26.74 ± 2.17

16. Chemical structures

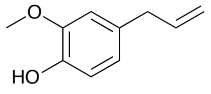
Eugenol

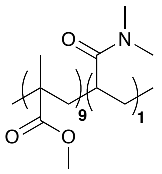
PA13

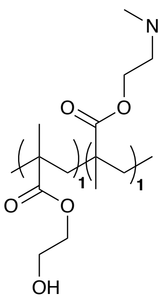
PA155

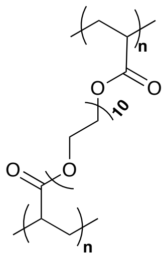
Poly(PEGDA-575)

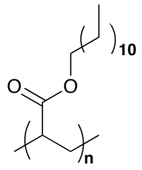
Poly(lauryl acrylate)

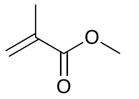
Methyl methacrylate

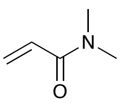
Dimethyl acrylamide

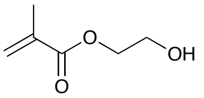
Hydroxyethyl methacrylate

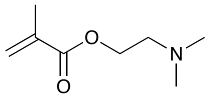
2-(Dimethylamino)ethyl methacrylate

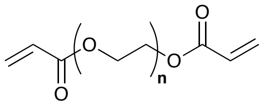
PEGDA-575
